# Creatinine clearance, reduced kidney function, and optimizing prescribing safety through practice feedback: a mixed methods study

**DOI:** 10.1093/fampra/cmaf062

**Published:** 2025-08-22

**Authors:** Su I Wood, Robbie Foy, Paul Carder, Stella Johnson, Duncan Petty, Sarah L Alderson

**Affiliations:** Division of Primary Care, Palliative Care and Public Health, Leeds Institute of Health Sciences, University of Leeds, Leeds LS2 9LN, United Kingdom; Division of Primary Care, Palliative Care and Public Health, Leeds Institute of Health Sciences, University of Leeds, Leeds LS2 9LN, United Kingdom; West Yorkshire Research and Development, NHS West Yorkshire Integrated Care Board, Bradford BD1 4AS, United Kingdom; West Yorkshire Research and Development, NHS West Yorkshire Integrated Care Board, Bradford BD1 4AS, United Kingdom; School of Pharmacy and Medical Sciences, University of Bradford, Bradford BD7 1DP, United Kingdom; Division of Primary Care, Palliative Care and Public Health, Leeds Institute of Health Sciences, University of Leeds, Leeds LS2 9LN, United Kingdom

**Keywords:** general practice, prescribing, quality and safety, feasibility studies, longitudinal studies, qualitative research

## Abstract

**Background:**

Kidney function declines with age, increasing risk of harm from raised blood levels of many medicines. Prescribing is often inappropriate for older people with reduced creatinine clearance (CrCl).

**Objective:**

To examine the feasibility and acceptability of providing performance feedback to increase CrCl calculation and coding and reduce potentially inappropriate prescribing.

**Methods:**

We delivered evidence-based feedback on CrCl coding and prescribing for common medicines requiring dose adjustment in renal impairment. This mixed-methods study in seven UK general practices collected data at three time points for evidence-based feedback (October 2021, December 2021, February 2022) and additionally pre-/post-feedback intervention. An institutional ethnography explored responses. We observed and conducted semi-structured interviews with primary care clinicians. We thematically analysed qualitative data, guided by Clinical Performance Feedback Intervention Theory.

**Results:**

Mean CrCl coding for ≥75s rose from 46% to 50.4% (difference 4.4%; range −10.5% to 14.7%). The number of patients with CrCl-associated inappropriate prescribing fell. We observed in 6 settings and interviewed 11 clinicians. Feedback engaged practices, was seen as important and allowed flexible action. All feedback cycle components were evident. Participants mentioned difficulties in remembering to consider kidney function, calculating and coding CrCl, recalling relevant medicines, and deciding appropriate dosing. Pharmacy teams were considered important facilitators in the response.

**Conclusions:**

Feedback on prescribing in reduced kidney function can encourage improvement but is not sufficient alone. Systematized CrCl calculation and coding may improve patient safety by facilitating decision support for prescribing, review, and audit/research. A rigorous, larger-scale effectiveness evaluation is likely to be feasible and acceptable.

## Lay Summary

Medicines are removed from the body by the kidneys more slowly as we get older, increasing risk of harm from raised blood levels of many medicines. Prescribing recommendations when kidney function is reduced are not often followed for older people. We examined how easy and acceptable it was to inform GPs on assessing kidney function and improving prescribing.

We collected anonymous data on kidney function and prescribing, five times over 11 months. We delivered three research-based reports. We noted change over time. We observed how reports were received and used in practice. Interviews with staff explored what they thought of the reports and any actions taken.

We observed a modest increase in recording of ‘creatinine clearance’ and that prescribing recommendations were followed more often. Participants said the topic is important. They liked and used the reports. However, remembering which medicines to check kidney function before prescribing is difficult. Pharmacy teams often provided practical support.

Reports on prescribing when kidney function is reduced can encourage change, but computer help is needed. We showed it is possible to provide routine data feedback, helping general practice teams to improve monitoring and prescribing in line with prescribing guidelines. However, more testing is required.

Key messages

- Kidney function declines with age, increasing risk of harm of many medicines.
- Feedback on prescribing reduced kidney function is feasible and acceptable.
- Systematized creatinine clearance calculation and coding may improve patient safety.
- This study demonstrated feasibility for a rigorous, larger-scale effectiveness evaluation.

## Background

Kidney function declines with age, increasing the risk of harm from many medicines. Prescribing recommendations when kidney function is reduced are often not implemented for older people in general practice [1, 2]. A cross-sectional study in 80 general practices found that kidney function was too low for recommended prescribing of study medicines in up to 40% of people aged ≥65 years, and up to 80% of people ≥85 years, even though 90% had a recent kidney function test recorded [1, 2]. A quarter of people aged ≥65 years are prescribed an average of 2 out of 70 different medicines where the kidney function is too low for recommended use [3]. Failures to adjust kidney-related prescribing harm older people, including a 40% increase in all-cause mortality [4] and a third of hospital admissions related to adverse drug reactions [5].

Estimated glomerular filtration rate, the commonly reported estimate of kidney function to identify kidney disease, can overestimate kidney function for prescribing decisions compared with creatinine clearance (CrCl) for older people, particularly for the very old [6–8]. Subsequently, patients inadvertently receive higher than recommended doses [8]. Use of estimated glomerular filtration rate inaccurately overestimates kidney function for up to 28% for those aged ≥65 years, and up to 58% for those ≥85 years [1]. Older people can have a normal reduction in kidney function with aging but without chronic kidney disease (CKD) or acute kidney injury (AKI), so need routine assessment of kidney function for prescribing. The British National Formulary (BNF) changed its recommendation in the Prescribing in renal impairment section in 2017 to state that CrCl should be used when prescribing for older people to estimate kidney function, particularly for those aged ≥75 [9]. In 2019, the UK Medicines and Healthcare products Regulatory Agency highlighted the need to use CrCl, as it is more accurate and useful for prescribing decisions for older people [10].

However, GPs have requested education and support as CrCl needs individual patient calculation; lack of automation means it is rarely coded in primary care electronic health records (EHRs), hindering identification of patients at higher risk [11]. Prescribing decisions needing assessment of kidney function are required when applying many national policies e.g. management of CKD, AKI, and diabetes, and the prescribing of direct-acting oral anticoagulants (DOACs). The combination of rising polypharmacy associated with multimorbidity, the aging population, and growing recognition of CKD has made prescribing increasingly complex.

Earlier work indicated that a feedback intervention, incorporating behaviour change techniques, can reduce potentially harmful prescribing [12, 13]. We assessed the feasibility of applying a similar feedback intervention to encourage calculation and coding of CrCl and support recommended prescribing for older people with reduced kidney function.

## Methods

### Design and setting

We undertook a mixed-methods feasibility study in Primary Care Networks (PCNs—groups of general practices collaborating to provide NHS services for registered practice populations; publicly funded universal healthcare coverage through taxation) [14] within a socioeconomically and ethnically diverse population in West Yorkshire, UK. To prepare for a larger scale implementation trial, we aimed to examine the feasibility and acceptability of the feedback intervention, and the methods used to test them [15].

We surveyed prescribing data at five time points before, during, and after intervention. We observed and interviewed primary care clinicians responsible for prescribing and quality improvement to explore the process and experience of receiving feedback on prescribing selected commonly prescribed medicines for older people with reduced kidney function. COREQ informed the methodology and reporting [16].

### Patient and public involvement and engagement

Our patient and public involvement and engagement (PPIE) group, familiar with implementation research [12, 13, 17], comprised seven people from diverse ethnic, occupational, and social backgrounds and varied levels of experience in healthcare commissioning and patient advocacy. They advised on the research question and protocol, topic guide, developed illustrative scripts to guide prescribers when talking with patients about medicines and kidney function (Supplementary data S1), and reviewed findings. We offered payment for PPIE time, in line with nationally recommended rates.

### Sampling and recruitment

We purposively recruited two PCNs (from four initially showing interest) to include one with experience of working on CrCl calculation and coding (PCN-A) and one not (PCN-B). All seven member practices gave informed consent to be included in the study and for use of practice anonymized data. The PCN research and pharmacy team leads invited staff on our behalf by convenience sampling. Those interested received the research information and, where consent was gained, were recruited for interview. We monitored recruitment against data saturation iteratively, to stop when three consecutive interviews elicited no new themes [18]. We planned to observe up to eight settings and recruit up to 20 interviewees. We offered participating practices and interviewees £100 each respectively to recognize opportunity costs.

### Intervention

We emailed evidence and theory-informed feedback reports to PCN leads, practice GPs, and pharmacy teams and posted to practice managers, in October 2021, December 2021, and February 2022, in the “Improving Prescribing in Renal Impairment” intervention (TIDieR Statement: Supplementary data S2). Practice-specific, comparative feedback reports presented the overall level of CrCl coding for the study medicines prescribed to patients aged ≥75 in the previous 8 weeks (example report: Supplementary data S3). DOACs, antidiabetic medicines, and antibiotics with BNF and licencing recommendations when kidney function is reduced [19, 20], were selected for risk, guideline recommendation, and prescribing pathway (Table 1).

**Table 1.** Medicine groups included and selected medicines used in the study.

Medicine group	Why chosen as an example for this study	Medicine examples with prescribing recommendations when kidney function is reduced (BNF, drug licence)	Number (%) of the study population (patients ≥75 years) prescribed the study medicines at the start of the study
Direct-acting oral anticoagulants (DOACs)	DOACs all have recommendations for use and dosing when kidney function is reduced. There is a high risk of bleeding if blood levels are too high. The BNF and guidelines state the need to use creatinine clearance for kidney function estimation	Apixaban 5 mg	124 (1.71%)
		Dabigatran any	3 (0.04%)
		Edoxaban 60 mg	36 (0.50%)
		Rivaroxaban 20 mg	222 (3.07%)
Antidiabetics	Diabetes can affect kidney function, and many antidiabetic medicines have recommendations for use and dosing when kidney function is reduced. There is a risk of e.g. hypoglycaeimia if blood levels are high; lactic acidosis with metformin. Most gliptins need a reduced dose when kidney function is reduced, except linagliptin. Many diabetics are managed by nurses who are not prescribers— staff not trained as prescribers might have different skills, knowledge, and behaviours.	Metformin any	500 (6.90%)
		Alogliptin 25 mg	27 (0.37%)
		Saxagliptin 5 mg	1 (0.01%)
		Sitagliptin 100 mg	33 (0.46%)
		Canagliflozin any	15 (0.21%)
		Dapagliflozin any	6 (0.08%)
		Empagliflozin any	15 (0.21%)
Antibiotics	Acute prescribing might entail different behaviours. Recommendations in reduced creatinine clearance need to be assimilated with the many antibiotic prescribing initiatives; Nitrofurantoin is ineffective at low creatinine clearance, and higher risk of pulmonary, hepatic, haematological, and gastrointestinal adverse effects. Tetracyclines can worsen kidney function.	Nitrofurantoin any	173 (2.39%)
		Tetracyclines any	10 (0.14%)

The reports presented data on CrCl calculation and coding for each study medicine. They were informed by evidence-based suggestions for effective feedback [21] and incorporated theory-based behaviour change techniques such as peer comparison and goal setting [22]. The feedback advocated coding CrCl with review of prescribing and suggested options for action whilst remaining non-prescriptive and leaving responses to practices’ discretion.

### Theoretical framework

Clinical Performance Feedback Intervention Theory (CP-FIT) informed our data collection and analysis (Supplementary data S4) [23]. CP-FIT is the first comprehensive theory on the conditions for optimal audit and feedback [23]. We compared the context and operation of the feedback reports in practice against CP-FIT to identify challenges and facilitators to implementing change in practice. We used CP-FIT components in developing questions to enquire about how PCNs and practices responded to and acted on the feedback.

### Data collection

For quantitative data, we tested feasibility by assessing required data accessibility, analysability, and utility.

We developed searches to be run in The Phoenix Partnership SystmOne EHR [24]:

- Number of patients aged ≥75 years prescribed the study medicines with CrCl coded in their record in the previous 2 years (extended from the standard 12 months to allow for COVID and test-tube shortage delays).
- Variables to calculate CrCl equation (BNF) [9] using age, sex, weight, height, serum creatinine in the previous 2 years, for patients aged ≥75 years prescribed the study medicines.

A clinical commissioning group data analyst ran the searches centrally at five time points: 3 months before intervention delivery (07/21), 1 week before each feedback report delivery (10/21, 12/21, 02/22), and 2 months after the final report (04/22). They provided an anonymized aggregated data set for analysis.

For qualitative data, an institutional ethnography explored how the feedback fitted into ways of working, and how, or whether, the ‘text’ (feedback reports) influenced healthcare [25, 26]. Observations and interviews were conducted by S.I.W., a post-doctoral pharmacist with primary care qualitative research experience (S.I.W. had worked with PCN-A in the past). Field notes recorded observations, and *ad hoc* conversations with staff in the field, following the trail from receipt of feedback reports through to any actions taken over the intervention period at PCN and practice level. We conducted semi-structured interviews on-line or face-to-face lasting 20–30 min. Interviews were audio-recorded and transcribed verbatim. Field notes were made immediately after each observation period and interview. The 11 CP-FIT feedback cycle components informed the guide for interviews and analysis (topic guide, Supplementary data S5) [23]. The first interview was reviewed as a pilot; no changes were made, and it was included for data analysis.

### Data analysis

CrCl code data were summarized graphically. We calculated CrCl using Microsoft Excel, and the number of patients requiring dose reduction or change in medication was analysed. We assessed the feasibility of a larger-scale statistical trend analysis. Whilst this study was not powered to test significance, we observed the quantitative data to assess whether a larger study would be warranted.

We analysed observation and interview data inductively considering CP-FIT [23]. We used a reflexive thematic analysis step-by-step approach of reading, coding, and identifying themes [27]. Whilst CP-FIT guided the analysis, we remained open to other notable findings. S.I.W. and S.L.A. independently coded a transcript for emerging themes and then compared coding and resolved discrepancies. The remaining transcripts were coded independently by S.I.W., with regular discussion with S.L.A. on emerging findings and themes. We generated overarching themes, combining and comparing codes charted in Excel, mapping how codes related, and looking for negative cases, to examine how practices received and acted on feedback reports.

All research team members were involved in data interpretation discussions. We used “following a thread” [28, 29] as an iterative process of data interrogation to interweave the findings that emerged from each dataset.

## Results

All seven practices from two PCNs participated and received feedback. Both PCNs had a pharmacy team providing services across all practices.

### Quantitative findings

At the first data collection, 1116 of 7253 patients aged ≥75 (15.4%) were prescribed one or more of the selected medications. Most had a serum creatinine recorded in the previous 2 years (range 95.2%–98.0%), whilst fewer (67.8%–76.4%) also had a recent height and weight recorded to allow calculation of ideal and adjusted body weights for CrCl estimation (Table 2).

**Table 2.** Variable and creatinine clearance code data for patients taking one or more of the study medicines.

	Pre (Jul 21)		Report 1 (Oct 21)		Report 2 (Dec 21)		Report 3 (Feb 22)		Post (Apr 22)	
Number of patients taking one or more of the study medicines	1116		1191		1202		1170		1169	
Number with a recent serum creatinine	1094	98.0%	1146	96.2%	1156	96.2%	1114	95.2%	1137	97.3%
Number with recent serum creatinine and weight	1034	92.7%	1046	87.8%	1060	88.2%	1016	86.8%	1044	89.3%
Number with recent serum creatinine, weight, and height	853	76.4%	820	68.8%	835	69.5%	793	67.8%	807	69.0%
Number with a recent creatinine clearance code on the record	532	47.7%	548	46.0%	539	44.8%	580	49.6%	589	50.4%

**Figure 1.**
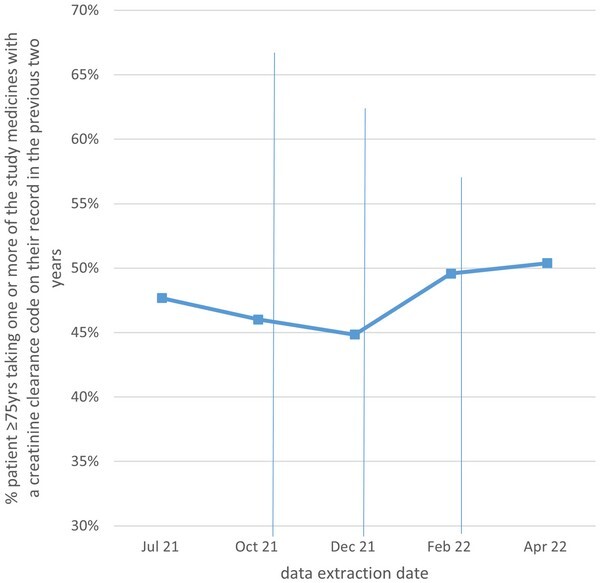
Percent patients ≥75 years prescribed one or more study medicines who have a creatinine clearance code in the patient record in the previous 2 years at five time points.

We observed an increased mean trend of 4.4% (−10.5% to 14.7%) of patients aged ≥75 with a CrCl code in the patient record in the previous 2 years prescribed one or more study medicines, from the beginning of the intervention to 2 months after the final feedback report (46.0%–50.4%; Fig. 1).

We observed that coding of CrCl seemed to increase in both PCN-A practices plus one in PCN-B but decreased in the other practices (Table 3).

**Table 3.** Patients ≥75 years prescribed one or more study medicines who have a creatinine clearance code in the patient record in the previous 2 years per practice.

		October 2021 (report1)			April 2022 (post)		
		Number prescribed one or more of the study medicines	Number (%) with CrCl code recorded in the previous 2 years		Number prescribed one or more of the study medicines	Number with CrCl code recorded in the previous 2 years	
PCNA	P1	357	189	52.9%	336	227	67.6%
	P2	154	59	38.3%	156	65	49.5%
PCNB	P3	215	84	39.1%	216	90	45.9%
	P4	71	30	42.3%	75	30	40.0%
	P5	193	91	47.2%	184	91	41.7%
	P6	105	53	50.5%	109	50	40.0%
	P7	96	42	43.8%	93	36	38.7%
Total		1191	548	46.0%	1169	589	50.4%

Coding of CrCl for DOACs averaged 95% before the start of the intervention, but only 33% for other study medicines. Coding appeared to increase (or remain 100%) for all medicines from the start of the intervention to the end of the study, except for tetracyclines where patient numbers were low (Table 4). We observed that fewer study medicines were prescribed where the CrCl was too low for appropriate prescribing (Table 4).

**Table 4.** For each study medicine, the % of patients aged ≥75 with a CrCl code on the record in the previous 2 years, and the number of patients with a creatinine clearance (CrCl) too low for appropriate prescribing.

Study medicines		October 2021—just before the start of the intervention					April 2022—2 months after the last feedback report was delivered					% change from the start of the intervention with CrCl code in last 2 years	Change from the start of the intervention with CrCl too low for recommended prescribing
		Number prescribed the example medicines	Number with CrCl code recorded in the last 2 years		Number with CrCl too low for recommended prescribing		Number prescribed the example medicines	Number with CrCl code recorded in the last 2 years		number with CrCl too low for recommended prescribing			
Direct-acting oral anticoagulants (DOACs)	Apixaban 5 mg	146	132	90.4%	3	2.1%	143	131	91.6%	0	0.0%	1.2%	−3
	Dabigatran any	6	6	100.0%	0	0.0%	6	6	100.0%	0	0.0%	0.0%	0
	Edoxaban 60 mg	36	34	94.4%	4	11.1%	31	31	100.0%	2	6.5%	5.6%	−2
	Rivaroxaban 20 mg	253	239	94.5%	17	6.7%	230	219	95.2%	16	7.0%	0.8%	−1
Antidiabetics	Metformin any	528	120	22.7%	9	1.7%	493	139	28.2%	6	1.2%	5.5%	−3
	Alogliptin 25 mg	26	6	23.1%	6	23.1%	30	12	40.0%	4	13.3%	16.9%	−2
	Saxagliptin 5 mg	1	1	100.0%	1	100.0%	1	1	100.0%	0	0.0%	0.0%	−1
	Sitagliptin 100 mg	35	8	22.9%	7	20.0%	31	14	45.2%	3	9.7%	22.3%	−4
	Flozins’ any	36	12	33.3%	5	13.9%	94	55	58.5%	6	7.1%	25.2%	1
Antibiotics	Nitrofurantoin any	203	56	27.6%	54	26.6%	185	53	28.6%	50	27.0%	1.1%	−4
	Tetracyclinces	8	2	25.0%	1	12.5%	10	2	20.0%	3	30.0%	−5.0%	2

The most recent CrCl code was not always up to date with the latest serum creatinine; CrCl recalculation showed the medicine was no longer appropriate in some cases.

### Qualitative findings

We observed in six settings: one on-line PCN clinical team meeting, one hybrid practice clinical team meeting, and four in-practice opportunities. One PCN worked closely together and held regular PCN-wide clinical meetings, which also considered prescribing feedback, whilst the other appeared to have no collaborative response.

We interviewed eleven healthcare professionals from six practices: five GPs (three with roles at PCN-level), five practice-based pharmacists (one pharmacy team lead), and one advanced nurse practitioner. We judged data saturation for themes around the feedback cycle as occurring after nine interviews (Supplementary data S6). Competing pressures on practices following the COVID pandemic, and study time restrictions, prevented us from continuing interviews to confirm saturation.

Themes evidenced all components of the feedback cycle (Supplementary data S4). Five themes emerged on: engaging PCNs, practices and pharmacy teams; flexibility in response; talking with patients; feedback is not enough for this topic; and constraints on action. More quotes are available in Supplementary data S7.

### Engaging PCNs, practices and pharmacy teams

The feedback intervention was reported and observed as being well received, with recognition that applying prescribing recommendations when kidney function is reduced is important for medicines safety and governance. Although DOACs were generally known to need kidney function estimation using CrCl, awareness for the other study medicines was limited, even for the pharmacists.

‘*I think it's crucial work, because if you look at high-risk drugs, whether it's Sotalol or digoxin or DOACs, it's quite crucial that the prescribing of those drugs and the doses are correct, so it's not optional work in my view, it's essential work, it's patient safety.*’ (D10: GP)

*‘Cockcroft Gault* [CrCl] *probably sits in a pharmacy world rather than a clinical world. I think people have got their head around eGFR, but they haven't got their head around Cockcroft Gault’* (D1: GP/PCN research lead)

There was evidence of further distribution of reports to more groups. Some requested the reports be sent to practice leads only, whilst others wanted direct emailing. No posted paper copies were seen, although some had printed copies from the email.

In both PCNs, the pharmacy teams were integrated within practices and were seen as important in their response. Participants said all clinicians need to be aware for a timely reaction as the pharmacy team will mostly review patients every 6–12 months.

The feedback reports were perceived to provide a clear, succinct, “*eye-opening”* (D5: practice pharmacist) presentation of the evidence and opportunities to improve. Trust in the data, bimonthly delivery, and evidence of change in prescribing, reinforced confidence. The different medicine groups detailed in each report focussed attention, and it was requested if it could be continued with further key medicines over a year. The content of the feedback reports helped engage staff when discussed at team meetings. All reported that they believed awareness had been raised and attitudes changed about a subject that is not always known about or remembered. As this was a short study it was perceived by some there had not been time for actions to start or to see the results of changes in behaviour.

‘*I am definitely thinking about it more, and I’m definitely doing that calculation* [CrCl] *more, which I wouldn't have done, without it*.’ (D7: Advanced Nurse Practitioner)

### Flexibility in response

Interviewees reported actions that fitted with existing resources and ways of working in response to feedback. These included setting personal goals, such as ensuring CrCl is coded and recorded every time it is calculated, and planning a quality improvement project on DOAC monitoring frequency. There was also evidence of organisational-level planning and action, such as CrCl calculation when pathology results are received for older people, identification of at-risk patients by practice pharmacists, and in teaching. Some reported using case studies in their team for learning, and others identified resources for their team.

‘*I am a lot more keen on making sure I record the fact that I have done a CrCl check…that I actively now put that code into the notes so that is being recorded.*’ (D9: lead PCN pharmacist)

‘*I had asked the pharmacy team to work on…initially we wanted to hoover up the DOAC people, who we’ve missed, and then it was moving onto looking at metformin*.’ (D3: GP prescribing lead)

### Talking with patients

Interviewees reported difficulties talking with patients about kidney function and prescribing and welcomed the suggested scripts developed with our PPIE group (Supplementary data S1). Two participants suggested adding words not to use when talking to patients, such as kidney ‘failure’.

‘*Having that conversation with patients is really difficult, because it's talking to people about something that's so abstract to them, about their kidney function and it's a hard conversation to have*.’ (D4: PCN pharmacist)

### Feedback is not enough for this topic

Interviewees said, although the feedback was important and raised awareness, prescribers need system-level help to apply the prescribing recommendations, and further training. They reported that calculating CrCl is easy but needs to be remembered in the process. They requested automatic calculation and coding of CrCl when the pathology results come into the practice to provide a timely result that is available for prescribing decisions and to search for at-risk patients. They said prescribers need to remember to think about kidney function, know and recall which medicines are affected, and decide appropriate dosing, placing too many demands on mental capacity and time to apply in the prescribing process. Many participants called for decision support, including prompts and formulary flags.

‘*I think the main barrier is just making it easier for people to calculate it… and warned you the patient's creatinine clearance is this…there must be an IT solution*.’ (D3: GP prescribing lead)

### Constraints on action

Competing priorities, compounded by COVID pressures and blood test-tube shortages, constrained response to the feedback. Participants suggested that perceived difficulties in managing prescribing in renal impairment may result in avoidance. Anxiety was expressed at finding out about an aspect of prescribing and medicines safety if not previously aware, and if additional work was needed.

Participants said guidance was unclear, with participant D2 finding the nitrofurantoin information in the BNF confusing. A pharmacy team lead and a prescribing lead requested the EHR searches to enable the finding of at-risk patients, but found they involved too many steps to be practicable.

‘*I honestly think the main barrier is just the workload on primary care at the moment… If everyone had a bit more time, it's definitely an area I think everyone would like to focus more on*.’ (D5: PCN pharmacist)

## Conclusions

### Summary

Feedback on prescribing medicines for older people with reduced kidney function is feasible to deliver and acceptable to general practices. We demonstrated complete data extraction centrally for CrCl calculations from EHRs, and delivery of feedback reports, which would be required for a larger scale definitive evaluation.

We observed modest improvements in CrCl coding and appropriate prescribing following the feedback intervention. Participants reported that feedback engaged practices, was seen as important and allowed flexibility in action, and that pharmacy teams facilitated responses. They found proactively calculating and coding CrCl relatively straightforward but remembering to consider kidney function, recall which medicines are affected, and decide appropriate dosing was difficult.

We observed variable responses by medicine category and by PCN. Over 90% of DOACs had a recent CrCl on the record, where there is clear guidance on the need to assess CrCl. For the other study medicines there was much lower CrCl coding, despite most having a recent kidney function test. Although participants were aware of, and confident in, assessing CrCl for DOAC prescribing and review, they were less so for the other study medicines. This is likely to be similar for the many other medicines that have prescribing recommendations when kidney function is low. Coding appeared to increase for many study medicines, although most improved coding and prescribing was observed only in the PCN with prior education and experience. Participants in the other PCN stated they intended to make changes but found it took time to implement and for benefits to become apparent.

Our feedback intervention was largely consistent with principles suggested by CP-FIT, and feedback constructs were evident [23].

### Strengths and limitations

Our study has added to the evidence on prescribing feedback, lack of renal monitoring, and optimizing medicine use. We demonstrated feasibility of delivery within emerging PCN organizations. We included pharmacy teams, known to influence difficult areas of prescribing [30]. We gathered perspectives from a range of healthcare professionals with roles in prescribing and medicines safety. This is an early study to use CP-FIT, which illuminated achievement of progress though the feedback cycle. The ‘institutional ethnographic’ approach of observations allowed actual behaviour and process to be examined, and interviews accessed the attitudinal data to explore the feedback report intervention.

We acknowledge six study limitations. First, this was a small-scale study without a control group and insufficient data points to allow a formal analysis of trends. However, we have demonstrated feasibility and acceptability for a larger scale, rigorous effectiveness evaluation.

Second, COVID pandemic restrictions affected observations, interviewee recruitment, and interview time; however, we were still able to elicit rich data. COVID and the blood test tube shortage led us to set 2 years for CrCl coding and variables to calculate CrCl (standard: 12–15 months).

Third, one practice previously worked with S.I.W; by purposive recruiting, we explored that legacy. Despite this, coding was low and potentially inappropriate prescribing evidenced. That practice achieved action in a short timescale, whilst others took time to get going despite intention.

Fourth, study participants were self-selecting, thereby probably under-representing those more resistant to change. However, sampling included practitioners who had not received the reports and one who had not acted until the interview.

Fifth, we did not explore the relative impacts of feedback components. However, interviewees did refer to behaviour change techniques without prompting, such as goal setting and action planning.

Finally, S.I.W's role as a pharmacist, and the team's stake in demonstrating feasibility, might have biased our analysis. Nevertheless, we have attempted to report our methods and findings transparently and regularly reflected on our assumptions and findings as a team, ensuring negative responses were identified and considered throughout.

### Comparison with existing literature

The 2011 large UK cross-sectional survey in 80 practices (70 000 patients) found that, despite over 90% of people aged ≥65 prescribed the study medicines having a recent recorded kidney function level, prescribing outside recommendations for use in renal impairment was widespread [1]. The BNF subsequently added a recommendation to use CrCl for kidney function estimation in older people [9]. CrCl is now commonly used to guide DOAC prescribing in primary care; our study suggests awareness for the many other affected medicines is still low. Potentially inappropriate prescribing of medicines and guidance for medicines optimization are reported for people with CKD or AKI [31, 32].

Although our study was not designed to assess effectiveness, the observed 4.4% increase in CrCl coding is compatible with the median effect on clinicians’ compliance with desired practice reported in a Cochrane review and that audit and feedback generally leads to small but potentially important improvements in professional practice [33]. A similar feedback intervention study to reduce potentially harmful opioid prescribing in primary care found an impact at the population level, and that feedback was cost-effective [13]. A subsequent process evaluation found practices took time to create changes in processes to reduce opioid prescribing [34], suggesting potentially greater effects from delivering the intervention over a longer period than was possible in the current study.

Pharmacists can have an important role in PCNs and practices for medicine optimization and review. A CP-FIT case study [23] on the “Pharmacist-led information technology-enabled” study [30] showed all feedback cycle processes were successful, and why feedback targeting pharmacists was effective at reducing patients at risk of medication safety errors.

This study added to the evidence for similar non-prescriptive feedback allowing practices to respond in ways that fitted within their ways of working and resources [12, 13]. Standardizing the function and components of the intervention, but not the processes to reach the goal, allows the intervention to be tailored to local contexts [35].

CP-FIT worked well for assessing the feedback cycle in practice, as was found in a recent exploration to improve the impact of two national clinical audit programmes [36]. However, other theories might unpack more about embedding new work practices [e.g., Normalization Process Theory (37)], or explore behaviour change [e.g., Behaviour Change Wheel (38)] to inform development of effective decision support.

### Implications for research and practice

Our work suggests a feedback intervention can encourage change. We summarized the relevant data needed and have demonstrated feasibility and acceptability for a large-scale study to rigorously evaluate effectiveness.

Our findings suggest CrCl calculation and coding should be automatic at the point of pathology results receipt to be available for acute and repeat prescribing decisions, medication reviews, long-term condition reviews, deprescribing, and searching for at-risk patients. Decision support tools could then be developed to help prescribers apply the recommendations and enable point-of-care testing, audit, and research. Recorded kidney function in the patient record does not necessarily lead to appropriate prescribing when kidney function is reduced [39, 40]. Although participants believed the feedback had raised awareness and that there would be behaviour change, they requested reminders and decision support to assess CrCl when prescribing or reviewing medicines.

We have shown the feasibility of delivering audit and feedback in primary care to improve prescribing in reduced kidney function and that prescribers find it helpful. Given the BNF and Medicines and Healthcare products Regulatory Agency guidance for the need to assess kidney function using CrCl when prescribing many medicines for older people, and the risk of patient harm if not considered, helping prescribers to apply the recommendations when kidney function is reduced will reduce the risk of adverse drug reactions, particularly for the oldest and most frail patients.

## Supplementary Material

cmaf062_Supplementary_Data

## Data Availability

Data are available on request.
