# Supplementary material for: Creatinine clearance, reduced kidney function, and optimizing prescribing safety through practice feedback: a mixed methods study
Source: Fam Pract. 2025 Aug 22;42(5):cmaf062. doi: 10.1093/fampra/cmaf062 (PMC12964551; doi:10.1093/fampra/cmaf062)

**Supplementary data S6: Data saturation plot: cumulative themes per Clinical Performance Feedback Intervention Theory (CP-FIT) feedback cycle component. No new themes were elicited after interview D9**

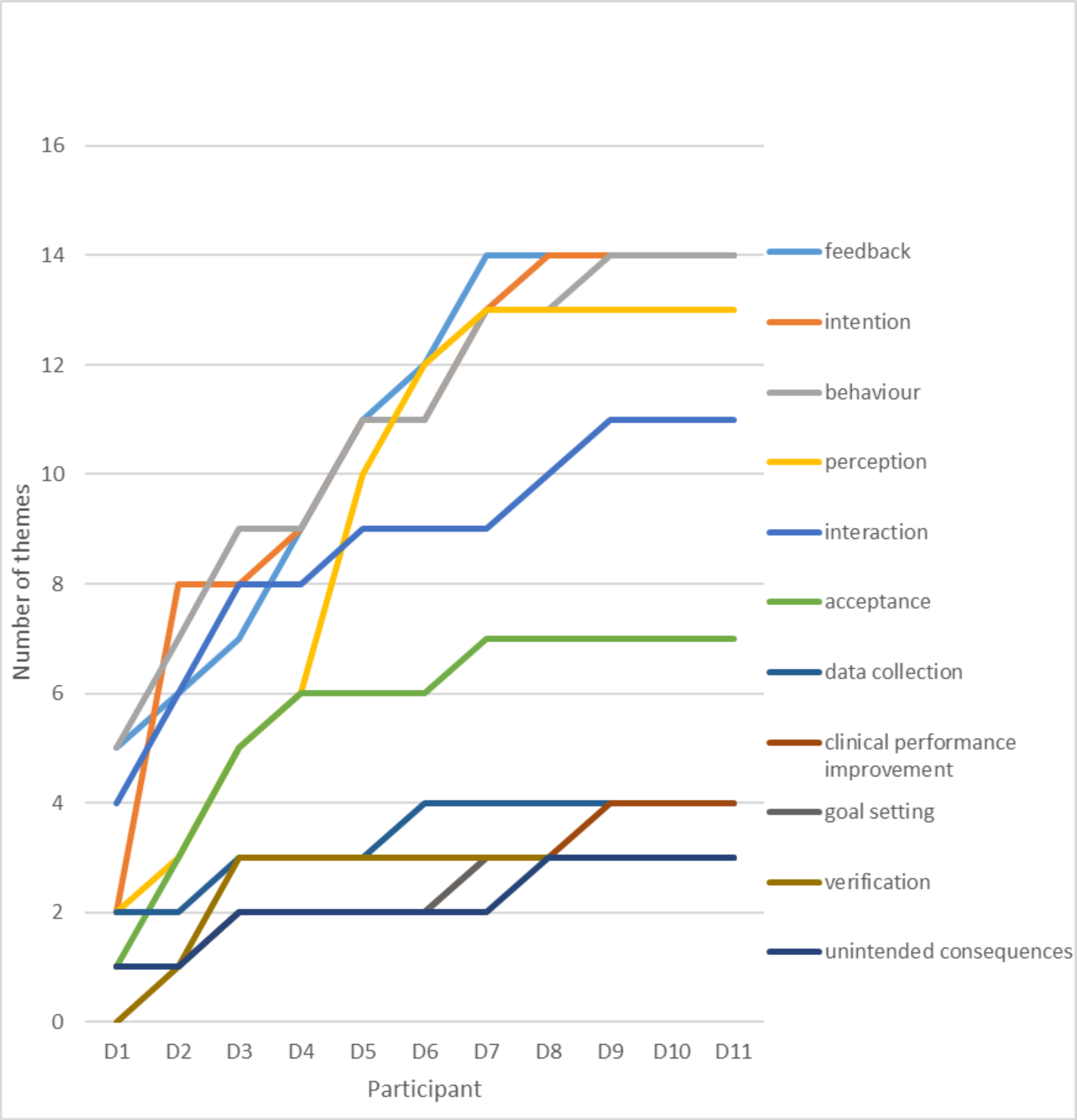

Supplement: cmaf062_Supplementary_Data [file cmaf062_Supplementary_Data.zip › Supplementary data S6.pdf]
