# Supplementary material for: Creatinine clearance, reduced kidney function, and optimizing prescribing safety through practice feedback: a mixed methods study
Source: Fam Pract. 2025 Aug 22;42(5):cmaf062. doi: 10.1093/fampra/cmaf062 (PMC12964551; doi:10.1093/fampra/cmaf062)
Supplement: cmaf062_Supplementary_Data [file cmaf062_Supplementary_Data.zip › Supplementary data S7.pdf]

Supplementary data S7: qualitative data all quotes

Engaging PCNs, practices and pharmacy teams

'*There's some really interesting stuff in here and I feel like after this interview I almost want to email my colleagues the key points that I've got from this, just some simple points that I could send to them in a couple of line email.*' D2

'*And I suppose it's helpful for us as an organisation moving forward because we're obviously two organisations that have come together and* [GP Practice name] *perhaps didn't have such a, that emphasis on creatinine clearance beforehand*.' D3

'*Yeah, making a very clear point about the creatinine clearance and how that differs from eGFR and you know the graph in particular was very, very handy you know just showing how different you know patients over 75 you know their values can be really, so it was quite eye-opening really, yeah*.' D5 (pharmacist)

'*We'd had a discussion actually before everything came out about sort of creatinine clearance, eGFR and to be quite honest, there was quite a lot of confusion with that with our team and we'd had a discussion about it and everything, but yeah the reports really put things into perspective and cleared up a lot of doubts and questions that we had, so yeah, yeah we found it really useful, yeah… I think as pharmacists we're more clued up really with this sort of area than other people and we, even we had sort of confusion about it so that's, so yeah and obviously other sort of areas, other healthcare professionals might even have you know further difficulty and further confusion.*' D5 (pharmacist)

Seen as important; trusted

'So I think the whole thing about renal function is really important to raise.' D1

'*Actually there's clearly room for improvement for Nitrofurantoin which is a very commonly prescribed antibiotic particularly in older people*.' D2

'*I think getting that data is helpful definitely. I think it's really important from a governance perspective that we do*.' D3

'*It's really sort of made me understand the topic a lot better and especially when carrying out sort of the medication reviews... I've had a few recently where I've had to reduce sort of diabetic medicine because I've checked like the creatinine clearance and things so, and sort of during, I think during our sort of structured medication reviews that we're going to be focusing on longer-term I think they're mainly going to be targeting sort of high-risk, majority elderly sort of patients and I think it has definitely brought to the front of my mind.'* D5

'*The kidney function, the creatinine clearance,... It's not a widely known thing anyway*.' D6

'*I think I found it useful mostly as a reminder, that I saw it, saw that we weren't doing great, and then would do something about it maybe for a week or two, and then forget, and then it would come through and I'd go oh heck we're not doing any better, or perhaps slightly worse, and so it would be, reminded me and reinforced for me to sort of do something about it*.' (D7)

'*I think there's the obvious ones that you straightaway sit there go oh dear and things like that, but there were others in there that I, that you know, I hadn't thought about quite as much, and I maybe thought, oh you know, I kind of knew that, but I wasn't really using that in every day practice, so that was useful as well*.' D7

'*You need to feel that I guess it's good practice to be doing this, and you know, so when you improve you sit there and feel good about it, because you know that you are providing better quality care for achieving that*.' D7

'*On acutes I don't think I've seen anyone note their renal function when it's been prescribed from the notes, from my memory, I don't prescribe them, but from my memory I don't think I've seen anyone do a CrCl alongside a nitrofurantoin so that's probably a big learning point*.' D8

'*I think it's crucial work, you know, because if you look at high-risk drugs, you know, whether it's Sotalol or Digoxin or NOACs, it's quite crucial that the prescribing of those drugs and the doses are correct, you know, so I suppose it's not optional work in my view, you know, it's essential work, it's patient safety.*' D10

'*eGFR is very clearly not working particularly in that older population, particularly in the AF population where they've invariably got two or three other long-term conditions*.' D11

Getting the feedback reports to those who need to see them

Emailed out to all GPs and pharmacy team leads; evidence of being distributed further (D7); no sign of the paper copies sent.

'*Emails are not always checked firstly, then they're not always received and when they are received they're not always read. Because some people have very, very busy inboxes and they prioritise some emails over others*.' D9

Gatekeepers

'*Everyone's very busy and we tried to, in some ways try and slightly protect our team from, to try and streamline where stuff is going because we used to get loads of stuff coming into all GPs…I personally think it probably is better to just go to, probably to a couple of lead, lead people in the practice*.' D3

'*If I had the report sent directly to my email it would be something I'd look at as it potentially would be fresh in my mind as to what kind of things we need to be addressing and where we're lacking in that particular site*.' [not forwarded the reports by the pharmacy team lead] (D6)

'*I think that it probably went out, you know, we have lots of groups, so I think because I would be, there's a GP and ACP group, so probably would have come out through that I would think, I'd say through that*.' (D7)

'*I suppose if it came from the surgery I kind of know everything that comes from the surgery because I need to be aware of anything that's happening immediately that I need to deal with, that [name] or anyone needs to tell me, I'm always kind of…so when he sent me your email I noticed it straightaway, but if it comes from kind of an influx of loads of other emails that I might miss*.' D8

'*I'm more alert to if there's something comes from the surgery, but if it comes from [PCN pharmacy team] I would still, you know, if my managers at [PCN pharmacy team] sent me something I would definitely look at it, but I would be cautious maybe if there was a, you know, … I think in December I*

was confused and I got the email, I wasn't sure what it was about… if it's from someone that I don't know.' D8

Pharmacy teams important

'*Then that's obviously reaffirmed it within the pharmacists' mind as well that it's, I mean I think they're already well aware of it but it's helpful*.' D3

'*They're* [pharmacy team] *becoming more and more integrated within our practice and we've got two main PCN pharmacists at the moment. I know that they are, they're struggling I think with staffing as well*.' D3

'*So with the pharmacy team yes it's absolutely understood and we've talked about it lots of times, we have a weekly meeting, and it's something that comes up a lot in the fact that we need to look, in certain patients we need to look at their creatinine clearance, and I think probably since everyone's even more aware of that, the fact that with certain patient populations we need to look at that rather than the eGFR*.' D4

'*I think in going forward definitely pharmacists are the, are a group that you can target to try and enforce it and improve things certainly*.' D5

'*As the prescribing team, as a pharmacy team, we tend to deal with anything related to medication, we actively take part in all like the QOF searches, the safety alerts, the NSAID prescribing safety, the sort of monitoring of medications as well, so everything like that falls into our remit as well*.' D6 (Pharmacist, PCNB)

'*I think in our organisation I would definitely say it would be more efficient for it just to come to the pharmacy team and then the recommendations of it to go outwards*.' D9

'*The problem is that the pharmacy team will only have a review every 6 or 12 months and then in-between the U&E will change, the creatinine will change, the patient will change, the drugs will change, so I suppose that's only useful for repeat prescriptions and that's only useful every 6-12 months*.' D10

Feedback report

'*I think we're often innately very competitive, so seeing data that says either that you are not in the middle, or at a good end of a spectrum, is often a nudge to do something about it*.' D1

'*And it's a great report, don't get me wrong [name], I think it's brilliant, I think it's a very good clear document and I don't think it could be presented any more succinctly*.' D2

'*I haven't questioned the data… Sometimes you do get a feel for it, some of the data quality reports, can tell when they're not quite right, but I didn't get a feel that those reports were unfairly reproduced'* D3

'*It's that everyone's busy and everyone gets lots of emails and if you can try and get it onto one or two sides even that's… Because our modern attention span, I think, is… Someone's more likely to look at that than to read a five page document. An initial summary and a kind of, I think the in vogue term*

is a deep dive, you know, something a bit deeper that people can click on I suppose if they want more information or tracking stuff down because some people will.' D3

'*I like how you focused on different things each time, rather than having everything in one, I think that's really useful*.' D4

'*I like how you focused on different things each time, rather than having everything in one, I think that's really useful.*' D4

'*I really liked them to be honest, yeah they were really sort of very sort of very well set-out, they weren't sort of overloaded with information but it was in a way where it was quite easy to interpret and sort of to very quickly work out sort of the key points really from it, and it put you at, it give you a very good idea of where you were at, sort of each individual practice you know compared to everyone else sort of locally, if you need, if you were doing well or if you needed to do any work*.' D5

'*Yeah, I had no sort of worries about it and it's probably, looking at the different, the data from the different sites as well it sort of, we sort of, we know that there's maybe a site or two in there that we think, we were sort of expecting it to be worse than others if you know what I mean, and sort of needing more work and things… we had no sort of queries over that*.' D5

'*I thought your reports were great, they were quite simple, they were well spaced out, yeah, it was easy to read, easy to look at, all the information on there was information that I wanted, in reality you have to be honest all of us skim read emails, and you know, and within skimming it there was enough information there for you to skim it and actually think it was beneficial for me to read that and, you know, which is what you want isn't it*.' D7

'*It was set out very clearly, and it was not too long, so it didn't take forever to read the whole thing, it was just set out in a nice way that I could read, in a ten, 15 minute break or, you know, in the evening, I could have, you know, scan my eyes over it. So it was quite easy to read, it wasn't bland*. *I think it was well thought out and well laid out*.' D8

'*So I really liked the feedback reports. Several reasons why. One is that, you know, we're in an environment in general practice where we have an unacceptable really turnover really of clinical staff just because we're using more long-term locums than we used to, we've not got as many partners, so one of the things that I liked about the reports was the fact that they didn't say "as you will already know" because that would have reduced their benefit to the team because when we send them out to the team they may not already know what we're on, what we're doing, so the fact that the report would always put in context of what we were actually looking at I thought was important*.' D10

'*I love colour banding, I really like the idea of, yeah, the traffic lights, I use it a lot for other issues, ECG interpretation, how we assess palpitations meant I like the idea of, you know, green, amber not so good, red we should be paying attention, and so I liked that that was reciprocated in the reports*.' D10

'*I really like benchmarking, you know, you've got to know where you are. If you're putting in a lot of work and you're still at the top, you know, the wrong end of the spectrum, it shows that maybe you're not directing it in the right way. So yeah, I'm very keen on benchmarking. Within a PCN I think it would be nice to know who's performing well and who isn't, mainly just so that you can ensure that within your group of colleagues good practice is being spread so that people who are achieving a lot can teach the people who may be struggling to work all this out*.' D10

The feedback raised awareness about a subject that is not always known about or remembered

Awareness raised

'I think stuff like 'Cockcroft Gault' is still, it's probably sits in a pharmacy world rather than a clinical world, and it probably ought… you know, I think people have got their head around eGFR, but they haven't got their head around 'Cockcroft Gault'' D1

'*A report like that shows actually there are some opportunities to improve. So I think having the reports allowed, I think allows you to see that there are always opportunities to improve.*' D1

'It's only been a short project but I think just having that data and then backing that up with a PBE session to re-highlight awareness to the whole team I think can only be a positive… I know it's been a lot of work so…good to raise awareness.' D3

'I was surprised with some of the drugs, and it probably shows my inexperience in my role, that I didn't know, well I did probably deep down, about Metformin and things like that, I did deep down but I don't do it as a matter of course at all.' D4 (pharmacist)

'*I thought they were really helpful actually, they were… with the DOAC stuff, the renal function and the DOACs, that I am really aware of and I think most pharmacists are, I think are aware of it when they're working, they always just double-check it, although there has been recently a few patients with the incorrect intervals of U&Es and stuff so that's something we're working on. But with the diabetic drugs, that was the one that I thought was actually really helpful to remind me to just check it, just check the creatinine clearance, because it might affect those drugs. So in that regard yeah it was actually really helpful to have a reminder and say oh it's not just the DOACs, there's other things that affect renal function, and doses change accordingly to that, so yeah I thought it was helpful*.' D8

'*There has been a positive impact from it to kind of make me aware, and I suppose your report prompted me to start checking the CrCl's for all the patients, not just when I've got a DOAC in front of me*.' D8

'*I think the reports will raise awareness certainly … the pharmacy team will only have a review every 6 or 12 months and then in-between the U&E will change, the creatinine will change, the patient will change, the drugs will change…so it also highlights that acute prescriptions will not be, you know, will not be captured by that pharmacist's background review process. So it will raise awareness*.' D10

Positive response, but takes time for action and change

All positive they have improved, or will in the future; attitudes changed

'*Even if you don't feel that you might have made much difference now, even if you've raised knowledge and started to change it that way, you may not change your numbers, but you may change people's attitudes to stuff. Even if you've raised a knowledge a bit of, so that actually future prescribing changes, rather than always changing what's, I think it would be positive*.' D1

'*Even if people have had a bit more awareness, that actually some of this, there are other ways of measuring stuff, but also that there's some drugs that they probably haven't thought about, that are more risky, that would be positive even though you might not see it as numbers, but attitudes and awareness I wouldn't underestimate that, because that percolates down to people, people teaching around them, so I would hope that that's changed, so that's a bit harder to measure unless you do an attitudes and confidence survey before you start.*' D1

‘*I think it does make change, it’s just it’s, it takes time doesn’t it, it’s just a very slow process*.’ D2

‘*Definitely… Well I had asked the pharmacy team, [name] to work on… So I think initially we wanted to hoover up the DOAC people, who we’ve missed and then it was moving onto looking at the Metformin side of things*.’ D3

‘*I mean from sort of our point of view as a pharmacist sort of group we sort of discussed it quite a lot at the time in our sort of weekly huddles that we have every Thursday so that was a big focus and a big, we tried to bring it to everyone’s attention and things*.’ D5

‘*So yeah, in my mind it’s definitely made a difference, yeah’* D5.

‘*I don’t think we did make a great deal of progress during it, and you know, might be times that we’re in that affected that, and so I think sometimes when you’re not making progress it can be quite easily something that you just stop looking at because you just don’t… that’s frustrating, but you know, we’re not getting anywhere*.’ D7

‘*We would have clinical meetings and possibly having [name – GP lead] as part of our team was a prompter, but I think we would have done anyway, we would sit there and go, you know, have you seen we’re not doing so well, bit of a push guys to kind of come on, or equally the reverse of patting ourselves on the back and saying actually we’ve done quite well, and we didn’t do any of that for this, because probably it's a short thing and, you know, we just hadn’t got into it…I don’t think in that short period of time we got to that point, but you’re probably right that was just length of time and the fact that I don’t think we necessarily had a local meeting to discuss it.’* D7

‘*I think for me there has* [been a positive response], *I am definitely thinking about it more, and I’m definitely sort of doing that calculation more, which I wouldn’t have done, without it, yeah, I’m definitely thinking about the drugs… It certainly personally has affected me, and I think, you know, although not in a massive way there is a difference to what I’ve done*.’ D7

‘*If I take the first report that came through, which was really, really good and it was about, if I'm correct, it was the DOAC prescribing was the first report. And it came through and I was like, well this is great but what do I do with this? And then it was having to speak to a few people to find out what it actually is. I think by the time we actually got it outwards it was almost the time for the second report to come through. So it just kind of, it had this reverse concertina effect where it started to bunch together, but then reports two and three we seemed to have a bit better response to*.’ D9

‘*I think everybody who was involved or had some exposure to the project has probably changed or adapted their practise to an extent and that's just the feedback that I get from people who I work with*.’ D9

‘*I think one of the things that we’ve noticed, yeah, one of the things that we’ve noticed [name] is a much better attention to NSAIDs and renal function*.’ D11

Flexibility in responding

PCNs and practices different ways of working

‘*We’re quite lucky really because sort of all [PCN name] is one organisation so you don’t have separate sort of GP practices within that sort of primary care sort of organisation so it does make things you know a lot more straightforward say, everyone works pretty much to the, or certainly*

moving towards working to the same way and the sort of differences between practices are becoming sort of smaller really, which is very, very handy.' D5

'*I suppose that might depend on the different Primary Care Networks, obviously they're quite, a bit like general practice they're quite heterogeneous aren't they in terms of how they filter information down? I know some Primary Care Networks will have like a, I don't know, three quarterly or 6 monthly meeting and then most people at the practice will go to that and then they'll talk about what's happening. I don't think that's happened in our PCN*.' D10

'*We have a very active research portfolio at [PCN name]…and we were more than happy to do so because a) it's about communicating to GPs about changing management, and b) it seemed like an important topic*.' D11

e.g. goals, behaviours reported

'*There was some really good stuff, I was having a, using some of the sort of sheets that you'd done when I was teaching yesterday to try and just get people to be more aware*.' D1

'*We've had, definitely we've had sort of queries, we get a lot sort of queries from GPs, nurses, everyone really and we do have quite a few on sort of renal function and whether things are appropriate and yeah, since I've, we've definitely sort of tried to just reinforce the creatinine clearance in sort of the over-75s*.' D5

'*I think we do try and be quite clear with that and also the eGFR creatinine clearance, when to use which one, we're also... We had pretty much another one that we presented in the meeting where that was raised and we all kind of looked at our learning and tried to work out when which should be used and we created a bit of an idea as to try and use it, use them both appropriately really*.' D6

'*I'm going to be doing like a QI project on DOAC doses, I don't know if it's going to be the big cohort of the full surgery because it has to be as part of the PCN pathway, so I have to do it in a certain time period, but it's something that we've discussed that I do want to look at it, because I've come across five or six patients now, just kind of missing that cut-off point where they'll drop into the next user needs check, so three monthly and they're on a six monthly, so they've kind of come up in the past week or two and I've noted it with the DOAC lead GP, and we're going to work together to try do some sort of audit*.' D8

'*From a personal point of view I know that I am a lot more keen on making sure I record the fact that I have done a renal check or creatinine clearance check. I can't say personally if I have started to actually check it more frequently, because I feel like I did do quite well before. But I know that I actively now put that code into the notes so that is being recorded.*' D9

'*The table and it's got all of the doses and what to do in various creatinine clearance levels? Yeah, we actually have that in our pharmacy hub, in the prescribing hub. We have that table printed off and on the wall and because it's something that we do often, it's essentially a very useful table to refer to*.' D9

'*And I've actually quoted some of the work that you've done in our new CKD pathway that we're doing at the moment. So this is about both diagnosis and ongoing management of CKD and how to define kind of AKI and who to do a creatinine clearance in. So we're using it as an organisation in some of the other kind of spoke off pieces of work that we're doing. So I think overall it has been a good thing for us to be involved in*.' D9

Want more feedback over time

‘*And it would be useful to maybe repeat that or repeat it in a certain length of time, say actually what’s happening, has the knowledge base changed and has your prescribing changed*.’ D1

‘*It’s often repetition isn’t it, as I’m sure you know from your experience, often you have to keep carping on about the same thing before the message finally sticks with everybody*.’ D2

‘*It is kind of looking at dripping those, breaking that up and dripping it in to people over a year or something like that with each report, with, I mean having, you’d have the crib sheet still, I think that’s a useful thing to have but possibly a useful way to raise awareness would be to trunk it up over a number of letters maybe*.’ D3

And education/ training

‘*We should try and put this into, make sure it’s embedded within training isn’t it*.’ D3

‘*The feedback was really good but I think the talk just sort of emphasised it really so yeah, I think it definitely sort of just brought it to everyone’s attention so yeah, I think the two together were good*.’ D5

‘*If people were encouraged to do it as a kind of day’s PLT, even if it’s half of their PLT in a month, I suppose every little bit helps because there might be people that look at that report and as I do, you know, keep it in my inbox unread and then eventually it goes down to the bottom and disappears. Until you have the annual clean out of the unread emails, you might not see it again. So I think it’s useful if the people read it but then whether they’ll always read it is difficult, so I suppose other means of trying to disseminate that information, you know, can also be, I suppose the more people hear about it in different formats and different things then the better, I suppose it sticks in your mind then*.’ D10

‘*I mean I think that needs education, I mean, you know, for people like me [name], you know, I grew up with creatinine clearance, I then got used to eGFR, then my own particular area of medicine creatinine clearance became much more important again but it wasn’t a particularly steep learning curve because I was just going back to what I used to do*.’ D11

Help with talking to patients

Scripts to help prescribers talk to patients – if patients know then can prompt prescribers. In addition would be good to add language to avoid.

‘*Language to avoid, so there are often words that really put, which really put, upset patients, especially so let’s say demented, is one thing, I always say to people, these are the words you don’t want to hear. And or ones that create fear, and if, and so you need to qualify them, so if you’re going to say the word ‘failure’, people think dialysis*.’ D1

‘*Talking to patients about their kidney function is often a difficult thing*.’ D3

‘*So I suppose some help about general conversations to have with patients, lines to use that aren’t, try and avoid distressing them probably is always going to be helpful. And a good resource to send*

them to about, you know, to learn more about it and there's leaflets that I send at the moment but I suppose if there was something more specific about drugs and that, that might be helpful. Probably an online, yeah, an online.' D3

'*Having that conversation with patients is really difficult, because it's talking to people about something that's so abstract to them, most of them anyway, about their kidney function and it's a hard conversation to have*.' D4

'*I suppose it's like whatever they talk in patient safety terms, it's Swiss cheese model and I suppose the more layers of cheese you have between you and the mistake the better off you'll be. So patient education might not be an unreasonable thing. I suppose the problem is that that doesn't apply to the over 75s because that's everybody [laughs] and whether they'll remember it, you know, is hard to say and how you spread that message*.' D10

'*I think it would be a useful extra filter if your patient knew. I suppose we do it for patients with reduced kidney function, we try and remind them, you know, before, well I try sometimes to remind them if they listen to a degree unless, so, you know, just make sure that when somebody prescribes something you have to remind them that your kidney function's reduced. But obviously that group's more easily identifiable than, you know, your reduced creatinine clearance patients. That's not as easy to do*.' D10

Feedback is not enough on its own, prescribers need system help to apply the prescribing recommendations

for prescribers to apply the prescribing recommendations

Calculating CrCl is easy to do, but routine calculation and coding of CrCl essential so available for prescribing decisions

'*Creatinine clearance is really easy to calculate, it takes 5 seconds doesn't it, you drop it down off the top bar and you click, you know, calculate. It's not, it's not a hard thing to do is it? And so, in terms of extra things to do before you prescribe it's not a hard task…* Creatinine clearance being automatically calculated, that would be helpful.' D2

'*That would make a huge difference actually [automatic calculation] because we actually have, for our heart failure patients, anyone who's sort of coded with you know heart failure, every time you go into their record it automatically carries out a CHA2DS2-VASc on them automatically and just as long as you save the record, it's there. So, even having that as a creatinine clearance for anyone…*' D5

'*If it was built in where the creatinine clearance was calculated as part of the blood tests themselves then maybe that might be more clearer*.' D6

'*I think you could see it [automatic calculation of CrCl] from two points of view, makes it easier, but then it isn't that easier, once you know where the renal calculation button is it's not that difficult to do [both laugh]. So it's, you know, so I mean you can have it there but you've got to look at it haven't you when you're prescribing and, you know, and I don't know how, I don't know how much difference there will be actually, I'm not sure it would make much difference*.' D7

'*That* [automatic calculation of CrCl] *would make a huge difference to the ability to implement a process*.' D10

‘*You know, the fact that the tools are there, automatic coding I think is a great idea*.’ D11

but more help for prescribers needed - Feedback not enough on its own; want prompts/ decision support; want to be able to search for at risk patients

‘*There’s certainly scope for commonly used medicines to make sure that all of them, for the ones which you’re more concerned about creatinine clearance, you have your Nitrofurantoin etc, you could easily write that on the formulary version of the medicine on SystmOne… and if another safety net within that is commonly used medicines that are an issue have a little guide attached to them I guess it’s just another way of reducing any potential error I suspect. I guess that’s where I’m coming at it from, it’s just another way of trying to help the clinician make the best prescribing decision*.’ D2

‘*Becoming a bit more of a routine to be thinking about checking the creatinine clearance… I think making that a bit better integrated in to SystmOne would be a big help with that*.’ D3

‘*I think the main barrier is, it’s just the, it’s just making it easier for people to calculate it really and for it to, to come up and I suppose it’s looking at, it is looking at whether there is a bit more of an IT solution to those drugs that do rely on creatinine clearance of whether you could have something that worked in the background and warned you, you know, patient’s creatinine clearance is this… there must be an IT solution*.’ D3

‘I do think it would be useful in the top right hand corner to have like a symbol saying this is the creatinine clearance, so you could just see that straightaway.’ D4

‘*I wonder whether there’s anything that can be done within SystmOne to help with the individual drugs, you know, you get the pop-ups, I know everyone hates pop-ups…but there’s much more worthwhile pop-ups that you could saying have you thought about creatinine clearance or something like that*.’ D4

‘*But I think having some sort of searches, like sort of CCG-wide searches at practices…sort of having these searches where you’d go in and you’d sort of almost check it on an ongoing basis I think would just give that extra…to that to identify if anyone was on the wrong dose of you know DOAC or anything like that, it’s, I think that would be really handy and that would make a big impact really from our point of view*.’ D5

‘*I think there is something around just remembering, you know, so saying you’ve got, you carry those drugs in your head that sort of set off a lot, you know, sort of an ace or something like that, which automatically makes you go oh dear, you know, but I think you know, I think carrying all that round in your head which is useful to have you know, if there was some way to… I think having a, having some way of those drugs being flagged as something might help*.’ D7

‘*There isn't an automatic prompt for it… if it's done it needs to be done in a smart way that people will actually pick up on*.’ D9

‘*I think having it (CrCl coded) on the system is definitely a good thing, but then it is again one of those things that all of these things are on the system and people need to then use it*.’ D9

‘*So if the creatinine clearance is in the notes and that is what's used for perhaps OptimiseRx or even SystmOne itself to either do a pop-up or, and I'm sorry for drumming it down into so much detail, but for example on the acute prescription dialogue box, there is a massive grey space to the right. So on there, on every single prescription it could just have patient's creatinine clearance is X and it just makes you think about that all of a sudden*.’ D9

'*If you've got it right at the top, nice and clear, patient's creatinine clearance is X, well then you can then just, it just then prompts you to just consider, well is this drug appropriate for this creatinine clearance or not? And where can you find that information? Well there is a whole list of places that you can find it, including that table that you've just sent, or attached to the pharmacy team or anywhere else that they can go to find information*.' D9

'*I mean the problem is that you have too many mentally competing demands, it's the same in hospital but in general practice it's, you know, I would argue in some ways it's a bit worse because, you know, you're not doing the same thing over and over, your patient problem changes, the number of things you have to do changes, there's clinical process, there's administrative process, there's pathway process so, you know, it's not possible to remember at every single turn and every single drug that, you know, renal prescribing needs to be at the forefront of your thinking. And I go through spells, so the more recently I've seen a report or the more recently I'm thinking about it the more creatinine clearance checks I do, and then as time goes on then you start fading back a bit. And it's the problem of fatigue, you know, whether it's pop-up fatigue or a particular prompt, even the visual prompt, eventually you fatigue and you start, your brain stops noticing. And I think that's the problem, you know, it's finding a system*.' D10

'*So awareness is one thing but then you need a system in the background that will robustly present you with that prompt or present you with the information you need, which is the way forward really*.' D10

'*The ideal is of course that you have the pop-up box as we get now with interactions and problems that you get after you do a prescription with every prescription, you know, and that's obviously embedded into the data sheet I suppose for that particular drug loaded in SystmOne*.' D10

'*The formulary so, you know, it needs to be embedded at that level. But short of them being embedded in that level if you can code a creatinine clearance when the last U&E comes in, you know, that would be better than nothing. Of course the problem is you have to make sure the weight and the height is up-to-date and so there are lots of other challenges there but it's better than what we have now which is we have to remember to do it*.' D10

'*So there's awareness on one hand and then there's, I suppose there's the different types of solutions, whether they're, you know, passive solutions so, you know, you have, you run clinical reports and try and identify cohorts of patients that don't have it done, you know, it's a very resource heavy commitment because you have to have somebody run the reports, then you have to have somebody actually check the notes, then you've got to have somebody call those patients back and make changes, whether all practices and all PCNs will have the, I suppose the ability to do that or the time to do that… then of course there's active solutions, you know, solutions that try to present the prescriber with their necessary information at that time. And I suppose most of those are likely to be I would think electronic*.' D10

'*I think that developing formulary flags that say, you know "this drug is renally excreted, have you consulted a recent creatinine clearance?" would also be another way to do that, particularly if the system's automatically coding creatinine clearance so that they're already in the system*.' D11

'*We have a point of care test where every time you dosed an older patient you could know what their renal function is before you do the dosing so that you can make sure that's it's appropriate? But we can't do that with the technology we're using at the minute because the returns and the congestion in general practice would just prohibit it, but we've got to get better at dosing and looking after people*.' D11

Other barriers are too many competing priorities, too difficult, catching up after COVID and blood test shortages, patient anxiety, references not clear, or assumption that someone else is doing it

Too many competing priorities

'*I'm conscious that I have looked at this before but probably never, never as thoroughly as I have done when I read through it yesterday. And I don't know why that is really, maybe we're all busy and we get these emails and…*' D2

'*I think one of the barriers I would see is just during the day is really clinically busy so you need really access to information very, very quickly*.' D2

'I honestly think the main barrier is just the workload on primary care at the moment, I think that's the only thing, I think if everyone had a bit more time, they'd, it's definitely an area I think everyone would like to focus more on.' D5

'*I think back to pre-Covid and the time that we were given to do things, I think it would have been hugely impactful because we could have really focused on it, but I feel like now my time is so taken up with all these requests, and all these med reviews that need to be re-authorised, and I'm doing the creatinine clearance within those tasks, so they're kind of done quite quickly and for me to kind of maybe if I wanted to do an audit and see what, you know, the Metformin dose, is it correct, but the time to do that, I would love to do that, but the time to do that isn't there, so I'm having to do it as I go along*.' D8

'*I mean the problem is that you have too many mentally competing demands*.' D10

Too difficult, anxiety if weren't previously aware, highlights a lot of work to do

'*I suppose you do want people to treat older people with, and it's usually older people, but people with reduced renal function and not say simply it's all too difficult, and there isn't, you know, we're not going to treat your diabetes because actually we can't think of a good enough drug to use sort of thing, that the worry is that people then shy away from everything.*' D1

'*The only problem it causes is it highlights to people that don't necessarily know about it or perhaps practices who will then suddenly find they've got a lot of work that they need to do*.' D10

Catching up after COVID and blood test shortage

'Because of Covid there's a backlog of patients, although we're getting a lot better now, there's a backlog of bloods that hadn't been done since 2019, 2020, so there's not up-to-date bloods for me to rely on too much and I'm really hesitant to rely on really old bloods, but that's getting better now.' D8

Patient anxiety

'*If we were to make changes to something that someone has been on for a very long time, some patients are really reluctant to accept any change, and it could even just be something, or it could be*

the nitrofurantoin thing, some people who are on a cycle maybe they're very reluctant to make any changes to that because they might get symptoms, or you know, whatever they perceive you know, to happen after stopping something. Could be a Metformin dose reducing, or it could be anything, but it might be that change that could throw them off and make them think that something's, you know, or might worry them, or make them anxious.' D8

References not clear

'*I was checking the BNF and even on something as commonly prescribed as Nitrofurantoin it talks about eGFR, but it didn't really obviously give the creatinine clearance reference range. Maybe I've missed something there, but I was looking at that this morning, it's very clear on eGFR but not, I couldn't see any obvious creatinine clearance ranges… That would be a barrier, if you're going to ask clinicians to check dose against creatinine clearance, they need an easy to go to reference point for that and it may be that that exists and I just don't know where that is*.' D2

'We had a query over the calculator on someone, and removing the height, what do you think of that, because I'm struggling to get my head around, because I trust SystmOne with that number and I go with it.' D8

Assumptions – some assumption that as there was a pharmacist team doing the med reviews that this was being done,

'*The pharmacy team obviously do do those creatinine clearance checks, so I think our performance is probably a bit better maybe than, it's probably better than the average. But obviously even with that in place and that, you know, presumed clinician knowledge, there still were some patients that weren't on the correct dose or might not have had the creatinine clearance check, so obviously it's not a completely robust system*.' D10

'*I think the ones that slipped the net are probably the ones who are in-between the pharmacist reviews and sometimes that might be whether someone's added it to repeat prescription having been started in the hospital who I assume must have their own systems to check creatinine clearance but some of them do come through with the wrong dose*.' D10

'*I mean I think the pharmacists anyway are very aware of that. I suppose that would be an interesting fact, you know, how aware they are, which patients they screen, which patients they do creatinine clearance on, but I'm sure they'll have, I assume they will follow the recommendations in the BNF because I think they've obviously got very clear-cut, you know, repetitive process in their reviews*.' D10
