# Supplementary material for: Creatinine clearance, reduced kidney function, and optimizing prescribing safety through practice feedback: a mixed methods study
Source: Fam Pract. 2025 Aug 22;42(5):cmaf062. doi: 10.1093/fampra/cmaf062 (PMC12964551; doi:10.1093/fampra/cmaf062)
Supplement: cmaf062_Supplementary_Data [file cmaf062_Supplementary_Data.zip › Supplementary data S1.pdf]

**Supplementary data S1: suggested scripts developed with the PPIE group to use when talking with patients about their medicines and level of kidney function.**

As we get older our kidneys gradually work more slowly. This is normal.

Many medicines are removed from the body by the kidneys. If the medicine is removed more slowly, it would mean higher levels of medicine left in the body. This could mean you are more likely to *come to harm [e.g.. For DOACs— have bleeding].*

### Starting a new medicine

As your kidneys are working more slowly now, we need to start the *medicine* at a lower dose.

### At medication review or inviting for review after an audit

As your kidneys are working more slowly now, we need to:

- lower the dose of your *medicine* to give the same effect.
- change your *medicine* to XXX
- ask you to make an appointment for a medication review with our pharmacist/ a GP to review your medicines.

We will do regular blood tests to check your kidney function for any changes.
