# Supplementary material for: Creatinine clearance, reduced kidney function, and optimizing prescribing safety through practice feedback: a mixed methods study
Source: Fam Pract. 2025 Aug 22;42(5):cmaf062. doi: 10.1093/fampra/cmaf062 (PMC12964551; doi:10.1093/fampra/cmaf062)
Supplement: cmaf062_Supplementary_Data [file cmaf062_Supplementary_Data.zip › Supplementary data S2.pdf]

Supplementary data S2: TIDieR checklist for the 'Improving Prescribing in Renal Impairment' (IPRIM) feedback intervention

1. **Brief name: Provide the name or a phrase that describes the intervention**

Improving Prescribing in Renal Impairment for older people (IPRIM)

2. **Why: Describe any rationale, theory, or goal of the elements essential to the intervention**

Audit and feedback (A&F) aims to improve patient care by reviewing health care performance against explicit standards. Ideally, where a discrepancy is detected, changes are implemented at an individual, team, and/or service level. Our earlier work identified that prescribing recommendations when kidney function is reduced are not often implemented for older people in general practice. Kidney function declines with age, increasing risk of harm from raised blood levels of many medicines, so helping prescribers apply the prescribing recommendations would benefit a substantial at-risk population. Also, in 2017, the British National Formulary (BNF) updated their recommendation to use the Cockcroft and Gault formula (CrCl) as the preferred method for estimating kidney function for older patients. There is little evidence on the effectiveness of interventions to help prescribers assess CrCl when prescribing and reviewing medicines. We therefore developed a feedback intervention targeting CrCl calculation and coding, and appropriate prescribing of medicines for older people with reduced kidney function in general practice.

3. **What (materials): Describe any physical or informational materials used in the intervention, including those provided to participants or used in intervention delivery or in training of intervention providers. Provide information on where the materials can be accessed (for example, online appendix, URL)**

The intervention entailed general practices receiving a comparative and practice-individualised feedback report on the number of patients ≥75 years with a CrCl code in the previous two years, the number for whom we could not calculate CrCl (missing serum creatinine, weight, height, sex), the number with CrCl too low for recommended prescribing of selected commonly prescribed medicines, and prescribing in relation to all other practices receiving the reports. From report two onwards, comparison to own prescribing at the start of the intervention was included. Aggregated patient data for each practice were extracted from electronic health record systems and reports were sent within one week of extraction. Individual prescriber-level data were not available and no patient outcome data were included. The three selected medicine groups were direct oral anticoagulants (DOACs), antidiabetic medicines, and antibiotics, and included medicines had prescribing recommendations when kidney function is reduced in the BNF and drug licence. The reports presented the data as total patient numbers and percentage of patient population, and in a bar chart that highlighted practices in the same Primary Care Network. Reports incorporated evidence-informed behaviour change techniques, such as specific recommendations for action and an action plan to complete, and the 15 recommendations for audit and feedback, designed to enhance effectiveness.

4. **What (procedures): Describe each of the procedures, activities, and/or processes used in the intervention, including any enabling or support activities**

Three copies of the feedback reports were sent to each practice bimonthly.

5. **Who provided: For each category of intervention provider (for example, psychologist, nursing assistant), describe their expertise, background and any specific training given**

Reports were sent by the West Yorkshire Research and Development team on behalf of the research team at the University of Leeds. The reports were written by a pharmacist and research fellow in primary care and reviewed by the research team.

6. **How: Describe the modes of delivery (such as face to face or by some other mechanism, such as internet or telephone) of the intervention and whether it was provided individually or in a group**

The feedback reports were sent by email to all GPs, the pharmacy teams and the practice manager at each participating practice. They were also posted to each practice, addressed to the practice manager, from October 2021 to February 2022.

7. **Where: Describe the type(s) of location(s) where the intervention occurred, including any necessary infrastructure or relevant features**

All seven member practices in two Primary Care Networks received the feedback reports,

8. **When and how much: Describe the number of times the intervention was delivered and over what period of time including the number of sessions, their schedule, and their duration, intensity or dose**

Feedback reports delivered bimonthly from October 2021 to February 2022 with a total of three reports sent.

9. **Tailoring: If the intervention was planned to be personalised, titrated or adapted, then describe what, why, when, and how**

No tailoring was done.

10. **Modifications: If the intervention was modified during the course of the study, describe the changes (what, why, when, and how)**

No modifications were made.

11. **How well (planned): If intervention adherence or fidelity was assessed, describe how and by whom, and if any strategies were used to maintain or improve fidelity, describe them**

Intervention adherence and fidelity was not assessed.

12. **How well (actual): If intervention adherence or fidelity was assessed, describe the extent to which the intervention was delivered as planned**

Intervention adherence and fidelity was not assessed.
