# Supplementary material for: Creatinine clearance, reduced kidney function, and optimizing prescribing safety through practice feedback: a mixed methods study
Source: Fam Pract. 2025 Aug 22;42(5):cmaf062. doi: 10.1093/fampra/cmaf062 (PMC12964551; doi:10.1093/fampra/cmaf062)
Supplement: cmaf062_Supplementary_Data [file cmaf062_Supplementary_Data.zip › Supplementary data S3.pdf]

NHS Bradford District and Craven Clinical Commissioning Group

UNIVERSITY OF LEEDS

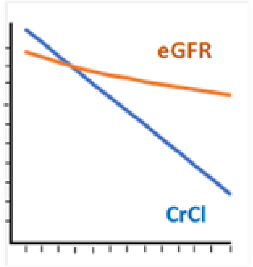

IPRIM
Improving prescribing in renal impairment

Can your practice review and improve prescribing for older people with reduced kidney function?

Dear colleagues,

In 2017, the **BNF** changed the recommendations for prescribing when kidney function is reduced.[1]

The **BNF** states that creatinine clearance should be used as the kidney function estimate when prescribing for older people (particularly those 75 years and older), in extremes of muscle mass, and for narrow therapeutic index and high risk drugs.

Kidney function reduces with age, albeit at varying rates. Older people may not necessarily have kidney disease, but the normal reduction in kidney function means we need to consider the level when prescribing the many drugs that are excreted by the kidney.

One study in Bradford primary care found that over 70 different types of drug were prescribed outside the recommendations to patients aged 65yrs and older.[2] An average of two drugs per person needed altering because of their level of kidney function.

Currently, it is not easy to apply the recommendations in the prescribing process, particularly when doing a medication review. We hope the three feedback reports, each focussing on different example drugs, will aid you to **'Think Kidneys'** when prescribing, and doing medication reviews, for older people. In this first report we will focus on DOACs; the second report will focus on antidiabetic drugs, and the third on antibiotics.

> "The use of drugs in patients with impaired renal function can give rise to problems for several reasons… many of these problems can be avoided by careful choice and use of drugs… I think we may underestimate how much harm we may be actually doing to patients by having them on the wrong drugs and wrong dosages."
>
> Andrew Lewington
> Consultant Renal Physician
> Honorary Clinical Associate Professor
> St James's University Hospital, Leeds

We invite you to review your practice's prescribing of medicines which have recommendations for altering use and dosing for older people, who are more likely to have reduced kidney function.

This is the first report for your practice. Please distribute to all prescribers, and your pharmacy team, within your practice and identify a time to discuss it at a practice meeting.

Yours sincerely,

Su Wood
Research Fellow, University of Leeds, on behalf of the research team

West Yorkshire Research and Development

Why should we review prescribing for older people with reduced kidney function?

Kidney function reduces with age, which can be compounded if there is also cardiovascular and/or kidney disease. A reduced kidney function means a risk of higher blood levels when drugs excreted renally are eliminated more slowly, and a reduced renal reserve to handle drug excretion when e.g. dehydrated.

Why creatinine clearance, Cockcroft Gault (CrCl), and not eGFR, for prescribing decisions?

eGFR and CrCl are not interchangeable.

The age factor in eGFR is exponential whereas for CrCl it is linear meaning that in older age there is a significant difference in the results from the estimates . Studies looking at major bleed risk, ADRs, hospital admissions, mortality,

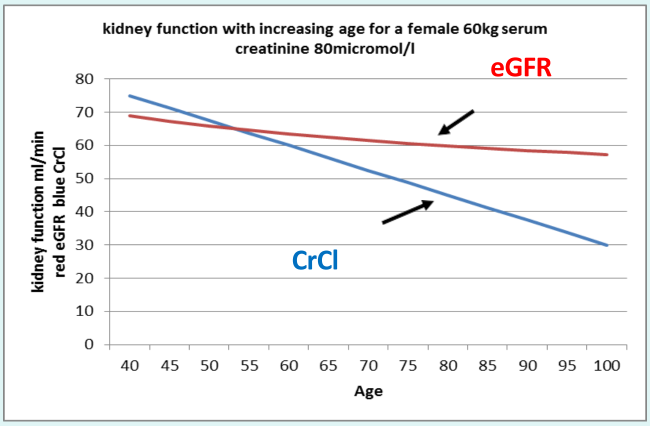

and drug blood levels[3] have shown that CrCl gives a better estimate for prescribing decisions for older people to reduce the risk of harm. Increasing old age increases the overestimation by eGFR meaning the oldest are at the most risk.

The **BNF** now states that creatinine clearance (CrCl) should be used to estimate kidney function for prescribing decisions for older people (particularly those 75 years and older), in extremes of muscle mass, and for narrow therapeutic index and high risk drugs.[1]

The MHRA Drug Safety Update Oct 2019 highlights this need to use CrCl for prescribing decisions in these high risk groups.[4]

Also in this report:

- **How your practice is doing** compared to other practices in the study on calculating and coding CrCl (**P3**).
- **What are your figures** for each of the study example drugs (**P3**).
- **Prescribing DOACs when kidney function is reduced, and monitoring** (**P4**) (report 2 will focus on antidiabetic drugs, and report 3 will focus on antibiotics).
- **Scripts to use when talking to patients** about their medicines when kidney function is reduced (**P5**).

Prescribing

Use the SystmOne renal calculator to calculate and code creatinine clearance: Creatinine clearance can be calculated on renal calculators e.g. SystmOne, EMIS, apps or on-line (e.g. MDCalc). The SystmOne calculator can be found at: **SystmOne > Clinical tools > renal disease calculations > save to the record to code**

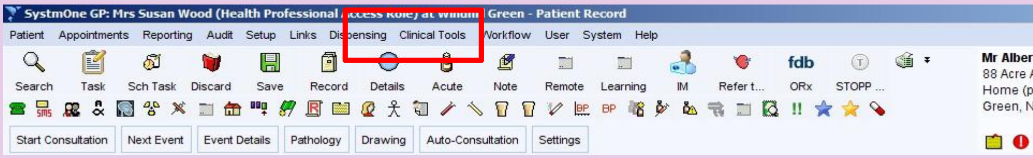

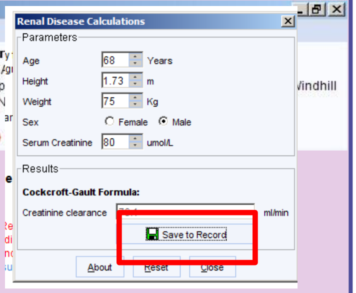

The calculator will use an ideal/adjusted body weight, or actual if lower, to give an estimated factor of muscle mass. You may need to amend for people with high muscle mass and use actual weight, e.g. body-builders, or e.g. amputees.

For prescribing recommendations in reduced kidney function see: the BNF - use the

How is your practice doing?

#PracticeName

The chart demonstrates:

- Your practice (black bar)
  - **Percentage of patients aged ≥75 years prescribed the example drugs in the last eight weeks with a creatinine clearance coded in the last two years (XX%)**
- A higher value indicates better clinical practice.
- The example drugs included are listed in the table below.
- The audit data does not exclude any patient groups.
- Participating practices (all in Bradford District and Craven CCG) range X to XX%).
- Red lines are other practices in your PCN

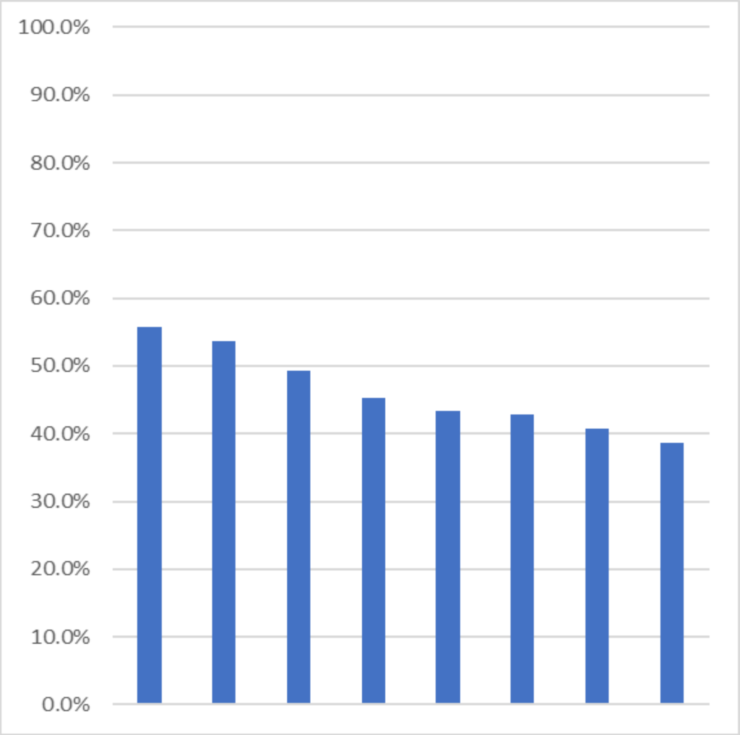

Your practice figures for example drugs prescribed for people aged 75 and older in the last 8 weeks

Example drugs	When CrCl is too low for recommended prescribing	Total number patients ≥75yrs prescribed the drug	Number with no CrCl code in the last 2 years	Number where we cannot calculate CrCl (need serumCr/weight/ height in the last 2 years)	Number patients with CrCl too low for recommended prescribing
**DOACs**					
apixaban 5mg	< 30 ml/min				
dabigatran any	< 30 ml/min				
edoxaban 60mg	< 50 ml/min				
rivaroxaban 20mg	< 50 ml/min				
**Antidiabetics**	**(report 2)**				
metformin any	< 30 ml/min				
alogliptin 25mg	< 50 ml/min				
saxagliptin 5mg	< 60 ml/min				
sitagliptin 100mg	< 45 ml/min				
‘flozins’ any	< 45 ml/min				
**Antibiotics**	**(report 3)**				
nitrofurantoin any	< 45 ml/min				
tetracyclines any	< 60 ml/min				

Direct-acting oral anticoagulants (DOACs) and reduced kidney function

All 'direct-acting oral anticoagulants' (DOACs) are excreted by the kidney, so when kidney function is reduced, there will be higher blood levels if the recommended reduced dosing is not adhered to, causing **increased risk of major bleeds.** The European Society of Cardiology (ESC) practical guide in 2013[5] clearly stated that kidney function should be assessed as creatinine clearance Cockcroft Gault. The MHRA have had reports of major bleeds resulting from incorrect dosing of DOACs not using CrCl.[4]

The table below summarises the dosing of anticoagulants for prophylaxis of stroke and systemic embolism in patients with non-valvular atrial fibrillation at different levels of kidney function.

CrCl >50 ml/min	**Any Anticoagulant** - no dose adjustment needed based on kidney function.
CrCl 30-49 ml/min	**Apixaban** 5mg bd or 2.5mg bd if at least two of the following: age ≥ 80 years, body weight ≤ 60 kg, or serum creatinine ≥ 133 micromol/L<br>**Dabigatran** 110mg bd if high risk of bleeding (suggest use of 'HAS-BLED' score to assess risk); otherwise 150mg bd.<br>**Edoxaban** 30mg od<br>**Rivaroxaban** 15mg od.<br>**Warfarin** INR dependant dose adjustment.
CrCl 15-29 ml/min	**Apixaban** 2.5mg bd<br>**Dabigatran** contraindicated.<br>**Edoxaban** 30mg od<br>**Rivaroxaban** 15mg od but caution - plasma concentrations significantly increased (average 1.6 fold) which may increase bleeding risk.<br>**Warfarin** INR dependant dose adjustment under expert advice and review.
CrCl <15 ml/min	No anticoagulant use recommended in general use, take expert advice

As **kidney function is likely to change over time**, there needs to be **regular kidney function tests and medication review.** Clinical Knowledge Summaries at www.cks.nice.org.uk/topics/anticoagulation-oral/management/ set out how each DOAC should be monitored. A summary of the recommendations for kidney function testing is given in the following example schedule of kidney function testing for DOACs based on level of kidney function (taken from Cambridge & Peterborough CCG guideline)

Drug	tests	Creatinine clearance	frequency
**Apixaban**<br>**Dabigatran**<br>**Edoxaban**<br>**Rivaroxaban**	FBC/ LFTs		every 12 months
	U&Es, weight, CrCl	>60 ml/min	every 12 months
		30-60 ml/min,<br>patient > 75 years or frail	every 6 months
		<30 ml/min	every 3 months (except dabigatran - contraindicated)
More frequent U&E's/LFTs advised where intercurrent illness may impact on renal or hepatic function.			

NB: knowing the CrCl will also ensure that a dose is not too low with increased risk of stroke.

What next?

Can you reduce the risk from prescribing for older people with reduced kidney function at your practice?

Make a plan about what your individual practice team members want to do, when and with whom. It may involve one or more of the following:

Think kidneys when prescribing for an older patient for the first time; is there a recent CrCl?

Think kidneys when doing a medication review for an older person; is there a recent CrCl?

Identify a practice **champion** who will lead on this work and look at relevant prescribing guidelines.

Consider **allocating records** for review within the team to the patient's usual GP or to a pharmacist for review and follow-up (if necessary) by usual GP.

Review your **progress** in light of further feedback we will send you later.

Talking to patients about their medicines and level of kidney function

Our Patient and Public Involvement group have helped us to draft some scripts that might help you talk to patients about medicines and their kidney function.

As we get older our kidneys gradually work more slowly. This is normal.

Many medicines are removed from the body by the kidneys. If the medicine is removed more slowly, it would mean higher levels of medicine left in the body. This could mean you are more likely to *come to harm [e.g.. For DOACs— have bleeding].*

Starting a new medicine **As your kidneys are working more slowly now, we need to start the** *medicine* **at a lower dose**.

At medication review or inviting for review after an audit **As your kidneys are working more slowly now, we need to:**

- lower the dose of your *medicine* **to give the same effect.**

- change your *medicine* **to XXX**

- ask you to make an appointment for a medication review with our pharmacist/ a GP to review your medicines.

We will do regular blood tests to check your kidney function for any changes.

#PracticeName team plan of action is to:

- What are we going to do (e.g. which risk factors would you like to review if any)?

- When are we going to do it (opportunistic, systematic, a combination or another time)?

- Who will be involved (GPs, pharmacist, administrative staff)?

Frequently Asked Questions

What is IPRIM (Improving Prescribing in Renal Impairment)?

IPRIM is a research project run by the University of Leeds in collaboration with Bradford District and Craven CCG led by Dr Su Wood, Pharmacist Research Fellow. We aim to help prescribers with applying prescribing recommendations when kidney function is reduced.

We are very busy. Why should we review our prescribing?

We know that practices are currently under a great deal of pressure and there are increasing demands within consultations. However, reducing risks in prescribing can prevent adverse events which would increase demands on your practice. CQC have recently been issuing urgent warning notices on medication monitoring - this review can be used as evidence for CQC.

Why are we looking at these drugs?

The example drugs have been chosen as they have been shown to be frequently prescribed in primary care for older people with a kidney function too low for recommended prescribing. [2,3] However, these are only a small proportion of the many drugs that have recommendations for how they are prescribed when kidney function is reduced—check the BNF, but remember you need to use the figures as CrCl, not eGFR , for prescribing decisions for older people; direct-acting oral anticoagulants (DOACs); patients taking nephrotoxic drugs (examples include vancomycin and amphotericin B); patients at extremes of muscle mass (BMI <18 kg/m^2 or >40 kg/m^2); patients taking medicines that are largely renally excreted and have a narrow therapeutic index, such as digoxin and sotalol.

Where do these data come from?

These data were extracted from SystmOne by the CCG in October2021. We have looked for CrCl codes in the previous two years to take into account the effects of COVID and the test tube shortage.

Recommended intervals depend on the drug and patient, but would normally be at least annual.

Why can't I produce the same numbers as the report?

It is important to remember that you may have changed patient care since we collected these data. SystmOne updates on a daily basis so it will not be possible to exactly replicate the figures in your practice feedback reports.

Report 1	Report 2	Report 3
August 2021	October 2021	December 2021

How often will I receive this feedback?

Practices will be re-audited every two months and an updated version of the feedback will be sent so you can see your practice's progress.

References:

1. BNF: prescribing in renal impairment. www.bnf.nice.org.uk/guidance/prescribing-in-renal-impairment
2. Wood SI, Petty D, Glidewell L, Raynor T. Are we over-dosing our elderly patients with renally excreted drugs in primary care? IJPP 2011;19 (suppl. 2):38
3. Wood S, Petty D, Glidewell L, Raynor DT. Application of prescribing recommendations in older people with reduced kidney function: a cross-sectional study in general practice. Br J Gen Pract. 2018 May 1;68(670):e378-87.
4. www.gov.uk/drug-safety-update/prescribing-medicines-in-renal-impairment-using-the-appropriate-estimate-of-renal-function-to-avoid-the-risk-of-adverse-drug-reactions
5. Heidbuchel H, Verhamme P, Alings M, Antz M, Hacke W, Oldgren J, Sinnaeve P, Camm AJ, Kirchhof P. EHRA practical guide on the use of new oral anticoagulants in patients with non-valvular atrial fibrillation. Europace. 2013; 15:625-651, DOI: 10.1093/europace/eut083
