# Supplementary material for: Creatinine clearance, reduced kidney function, and optimizing prescribing safety through practice feedback: a mixed methods study
Source: Fam Pract. 2025 Aug 22;42(5):cmaf062. doi: 10.1093/fampra/cmaf062 (PMC12964551; doi:10.1093/fampra/cmaf062)

Supplementary data S4: A summary of themes found relating to the Clinical Performance Feedback Intervention Theory (CP-FIT)[23] feedback cycle components for the study intervention.

1. Goal Setting
- See goals as relevant, credible, important. "It's crucial work…it's not optional, it's patient safety" (D10)
- Evidence of goals set by practices e.g. to improve DOAC monitoring (P1, D8)
- Evidence of goals set by individuals e.g. to record and code when checks CrCl (D9)

2. Data collection and analysis
- Confidence in the data – trusted, credible
- P1 and PCNB pharmacy team used case studies to present and discuss the issues
- There were requests for the searches to find the at risk patients but found too complicated; not currently easy to use – needs to have CrCl routinely coded to make straight forward.
- Wanted to continue longer e.g. for one year to cover more drug groups

3. Feedback
- The message was clear, succinct, relevant; shows opportunities to improve and how (D1)
- All liked the reports: didn't assume knowledge, language clear and simple, a good overview, benchmarking and Red/Amber/Green use good
- Liked the framing – focus on a different drug group each report.
- Most liked the length and content, "easy to get the key points" (D8); two wanted a short summary/ top-tips with more detail available for those that wanted it (D3, D4)

4. Interaction
- Reports were emailed to all GPs and the pharmacist teams in the two PCNs; sent from West Yorkshire Research & Development which was not always recognised.
- PCN pharmacy teams important to reach – they do all the medication reviews
- Emailed reports received and evidence of being forwarded on e.g. to D7 (Advanced Nurse Practitioner). Posted paper copies were not evident
- Gatekeepers: reports were not necessarily forwarded to the team e.g. pharmacists who wanted to see them. Some wanted reports to be only sent to a practice or PCN lead
- The two PCNs were very different: one only interacted at practice level, the other worked together as a PCN
- Team meetings stimulated interaction.

5. Perception
- "eye-opening" (D5)
- All thought it is important and impactful; important for governance
- Well received, useful; clear where work is needed
- Cleared up previous confusion, doubts, and questions
- Needs education

6. Verification
- Participants did not feel the need to check the data
- Difficult to search for at risk patients because of low CrCl coding
- Checking in the BNF is confusing

7. Acceptance
- Well received
- Fitted with understanding; trusted the reports
- More aware for DOACs, but not previously aware for other drugs
- Information and data in the reports used in teaching, and in a PCN CKD/AKI guideline
- Confusion about advice on system CrCl calculators raised by many participants

8. Intention
- Want to reduce risk and hospitalisation for patients
- Wanting to action, disseminate, have peer discussion.
- Main barrier is time and workload. Too many mentally competing priorities
- Want to do it, but don't think about it as much as should –
- Although it is easy to do if remembered, it would be good to have automatic calculation and coding of CrCl, but then need to have active support to apply when prescribing
- Need the system to help; need prompts and reminders; this would be a topic where a pop-up would be welcomed
- Talking with patients about this is difficult

9. Behaviour
- Pharmacy teams central to action in both PCNs; they have noticed more requests for review of medicines when kidney function is reduced
- Evidence of team discussion (pharmacy and wider clinical); work on specific drugs, use in teaching and guidelines; work to reinforce use of CrCl when prescribing for >75s; work to code CrCl when receive pathology results for older people; a quality improvement project started on DOAC monitoring as a result of IPRIM; table of recommendations used regularly as a reference
- Scripts for prescribers to use with patients are good – suggestions are to add what language NOT to use (e.g. 'renal', 'impairment'), and to provide a patient leaflet and/or a patient-friendly summary.
- Believes clinically improved personally, there is a difference in what he does (D7)

10. Clinical performance improvement
- All positive that have improved, or will in the future, personally and as a team; but may be patchy
- Thinks others have changed attitudes and practice; noticed better attention to kidney function and e.g. NSAIDs (which weren't included in IPRIM)
- Pleased to see work done showing in improved feedback (D3) vs frustrated that have worked to change but no improvement in feedback, maybe because too short a timescale (D7).

11. Unintended consequences
- Raised awareness

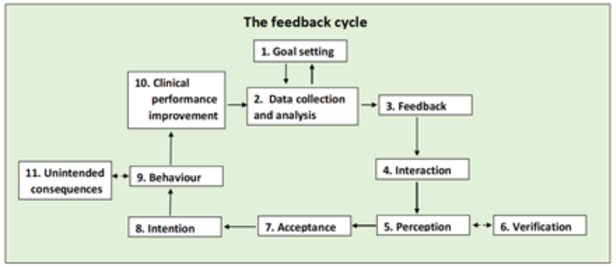

Supplement: cmaf062_Supplementary_Data [file cmaf062_Supplementary_Data.zip › Supplementary data S4.pdf]
