# Supplementary material for: Creatinine clearance, reduced kidney function, and optimizing prescribing safety through practice feedback: a mixed methods study
Source: Fam Pract. 2025 Aug 22;42(5):cmaf062. doi: 10.1093/fampra/cmaf062 (PMC12964551; doi:10.1093/fampra/cmaf062)
Supplement: cmaf062_Supplementary_Data [file cmaf062_Supplementary_Data.zip › Supplementary data S5.pdf]

Supplementary Material S5: The CP-FIT feedback cycle components [23] and associated interview questions

CP-FIT feedback cycle component	Definition	Interview question
1. Goal setting	CP-FIT hypotheses that feedback is more effective when the clinical performance standards are considered important and relevant to recipients' roles.	Are the standards of clinical performance clear in the feedback?<br>Are they clinically meaningful to you?<br>Are they something that could be managed by you/your team?
2. Data collection and analysis	Automated data collection and analysis processes are generally recommended. Manual collection and analysis are often hindered by a lack of time or skills.	Are you confident about how the data collection and analysis have been done?
3. Feedback	Current best evidence supports more frequent provision of feedback. Data should also be as recent as possible, which may enhance subsequent cycle components (Acceptance, Intention, and behaviour) and encourage identification of suboptimal performance.<br>Other relevant factors include problem-solving and action planning, i.e. helping recipients and introduce solutions to improve.	What is the feedback telling you? How useful is it?
4. Interaction	This component includes the method of delivery and how recipients interact with the feedback, e.g. is it delivered directly to clinicians or do they need to seek it out?	How do you get the feedback? Were the reports clear and easy to understand?<br>What happens to it in practice?<br>Has your practice shared the reports with your Patient Participation Group?
5. Perception	Feedback is more effective when it is user-friendly. Provision of a comparator (e.g. showing performance benchmarked against appropriate others) is considered to facilitate the perception, Intention, and behaviour components.	How well is the feedback understood, do you think? For you? For other members of your team?
6. Verification	A potential component between perception and acceptance where, if the feedback permits, recipients can explore the data underlying performance.	Can you interrogate the data further? And did you?
7. Acceptance	Acceptance is facilitated when recipients believe the feedback presents a true representation of their performance. Users are more likely to engage with credible feedback, which facilitates several cycle components.	Is there acceptance of the feedback? By you? By other members of your team?
8. Intention	Ideally, recipients form Intentions to take actions to improve performance in response to the feedback.	Has the feedback led to a planned response?<br>Have there been any barriers to action?<br>Is this an important topic? Where does it stand in your/the team's priorities?
9. Behaviour	Feedback that has been received, understood, and accepted will ideally be followed by a planned behavioural response. A distinction is made between patient-level responses, i.e. relating to the care of<br>individuals, and those at the organisational level with impacts across the wider healthcare system.	Has there been a behavioural response? Patient or organisation level?
10. Clinical performance improvement	Organisation-level behaviours are associated with greater clinical performance improvement potential as they enable multiple patient-led behaviours by enhancing the clinical environment in which they occur.	Have there been positive changes to patient care as a result of feedback?<br>Do you think there will be in the future?

11. Unintended consequences	CP-FIT acknowledges the potential for both positive and negative unintended outcomes of feedback interventions. Examples include improved recordkeeping, or manipulation of patient populations to artificially improve performance, respectively.	Have there been, or could there be, any unintended consequences as a result of the feedback?
